# Differential Proteome Analysis Identifies TGF-β-Related Pro-Metastatic Proteins in a 4T1 Murine Breast Cancer Model

**DOI:** 10.1371/journal.pone.0126483

**Published:** 2015-05-18

**Authors:** Misako Sato, Tsutomu Matsubara, Jun Adachi, Yuuki Hashimoto, Kazuna Fukamizu, Marina Kishida, Yu-an Yang, Lalage M. Wakefield, Takeshi Tomonaga

**Affiliations:** 1 Laboratory of Proteome Research, Proteome Research Center, National Institute of Biomedical Innovation, Saito, Osaka, Japan; 2 Department of Hepatology, Graduate School of Medicine, Osaka City University, Osaka, Japan; 3 Department of Anatomy and Regenerative Biology, Graduate School of Medicine, Osaka City University, Osaka, Japan; 4 Laboratory of Cancer Biology and Genetics, National Institutes of Health, Bethesda, Maryland, United States of America; University of Tennessee Health Science Center, UNITED STATES

## Abstract

Transforming growth factor-β (TGF-β) has a dual role in tumorigenesis, acting as either a tumor suppressor or as a pro-oncogenic factor in a context-dependent manner. Although TGF-β antagonists have been proposed as anti-metastatic therapies for patients with advanced stage cancer, how TGF-β mediates metastasis-promoting effects is poorly understood. Establishment of TGF-β-related protein expression signatures at the metastatic site could provide new mechanistic information and potentially allow identification of novel biomarkers for clinical intervention to discriminate TGF-β oncogenic effects from tumor suppressive effects. In the present study, we found that systemic administration of the TGF-β receptor kinase inhibitor, SB-431542, significantly inhibited lung metastasis from transplanted 4T1 mammary tumors in Balb/c mice. The differentially expressed proteins in the comparison of lung metastases from SB-431542 treated and control vehicle-treated groups were analyzed by a quantitative LTQ Orbitrap Velos system coupled with stable isotope dimethyl labeling. A total of 36,239 peptides from 6,694 proteins were identified, out of which 4,531 proteins were characterized as differentially expressed. A subset of upregulated proteins in the control group was validated by western blotting and immunohistochemistry. The eukaryotic initiation factor (eIF) family members constituted the most enriched protein pathway in vehicle-treated compared with SB-43512-treated lung metastases, suggesting that increased protein expression of specific eIF family members, especially eIF4A1 and eEF2, is related to the metastatic phenotype of advanced breast cancer and can be down-regulated by TGF-β pathway inhibitors. Thus our proteomic approach identified eIF pathway proteins as novel potential mediators of TGF-β tumor-promoting activity.

## Introduction

Breast cancer is one of the most studied cancer types and has a well-established molecular classification based on gene expression profiling [1]. Furthermore, immunohistochemical staining of primary tumors with anti-ER/PR, Her2 and Ki67 antibodies has been used to identify breast cancer subtypes for selection of therapeutics such as estrogen response modifiers, aromatase inhibitors and Herceptin that have shown some efficacy in shrinking primary tumors and prolonging patient survival. However, metastasis is still problematic in breast cancer, more than 80% of patient with breast cancer receive adjuvant chemotherapy, as it is not possible to predict the risk of metastasis development, and approximately 40% of the patients will ultimately relapse and die from metastatic disease [2]. Many aspects of the molecular etiology of metastasis are still not clear and the metastatic lesion develops in a very different microenvironment from the primary tumor. As a result, primary and metastatic lesions frequently differ in response to therapeutics, with metastases being much more therapy-resistant [3]. Therefore, to understand the disease at the metastatic level it is important to identify the active biological pathways in both the tumor parenchyma and microenvironment at the metastatic site, to provide leads for development of more effective therapeutic outcomes for patients suffering from later stages of the disease.

Transforming growth factor- (TGF-) is a pleiotropic growth factor and plays a dynamic role in both the tumor parenchyma and the cells of the tumor microenvironment [4]. TGF- generally acts as a tumor suppressor in the early stages of epithelial carcinogenesis and switches to a pro-oncogenic role later in disease progression [5,6]. TGF- overexpression in many advanced tumors correlates with metastasis and poor prognosis [7] As a result, approaches to antagonizing the TGF-β pathway have been developed, including a number of small molecule compounds have been developed that target the TGF- signaling pathway by binding to the ATP-binding pocket of TGF- receptor I kinase, preventing TGF-β-mediated downstream signaling events [8,9]. Both clinical and pre-clinical data show that the application of TGF-β antagonists successfully prevents or suppresses advanced metastatic disease in a number of preclinical models [7]. Nevertheless, further understanding of TGF-β biology in tumor progression is critical to avoid treating patients who still have TGF-β suppressive effects active in their tumors and finding specific surrogate markers of TGF-β signaling events involved in the cancer progression, is a high demand for an individual patient before initiating anti-TGF-β drug treatment.

The murine 4T1 breast cancer cell line was originally isolated by Fred Miller and coworkers at the Karmanos Cancer Institute in Detroit, MI. It was derived from a Balb/c mouse mammary tumor and has been extensively characterized for its metastatic properties [10,11]. It closely resembles triple-negative, basal-like breast cancer. When introduced orthotopically, 4T1 leaves the primary site and efficiently forms visible metastatic nodules in the lung; therefore, this model has been used for preclinical experiments for drug intervention [12]. A number of studies have shown that treatment with TGF-β antagonists can suppress 4T1 lung metastasis through combinatorial effects on multiple cellular compartments [13,14]. Recently, this mammary breast cancer progression model was analyzed to identify potential breast cancer metastasis-associated proteins using an iTRAQ-based quantitative proteomic method on the *in vitro* cultured cell lines [15,16]. This type of approach has been used as a tool for discovering cancer biomarkers and developing novel anti-cancer therapies [17,18]. We also believe that a proteomics approach is a useful advanced technology in monitoring the status of key factors of TGF-β signaling pathway in cancer progression.

In this study, we sought to identify protein profiles to distinguish the underlying TGF-β-specific biology that might be useful for developing effective therapeutic strategies. We hypothesized that the proteomic differences between cells in metastatic lesions with and without treatment with a TGF-β inhibitor would reflect the acquired TGF-β oncogenic activity characteristic of the later stages of tumor progression. To model treatment in the clinical setting, we used a small molecule TGF-β receptor I kinase inhibitor, SB-431542, to treat established orthotopic 4T1 mammary carcinomas. Importantly, for this study we surgically dissected out isolated metastasis nodules in order to examine the tumor proteome in the context of the metastatic microenvironment. We aimed to find any key factors associated with metastasis in response to TGF-β. We propose that such a global quantitative proteomics approach would identify TGF-β-related proteins that may serve as potential drug targets to block TGF-β oncogenic effects at the metastatic site but not influence tumor suppression.

## Materials and Methods

### Cell line and mouse experiments

The 4T1 cell line was cultured as previously described [19]. Briefly, cells were cultured in DMEM supplemented with 10% fetal bovine serum and incubated at 37°C in a humidified atmosphere containing 5% CO_2_ until 70% confluent. Cells were suspended as single cells, washed twice with PBS and then resuspended in DMEM for mouse xenograft implantation. Ten thousand 4T1 cells were injected subcutaneously into the second mammary fat pad of 6-week-old Balb/c female mice (Japan SLC, Inc., Shizuoka, Japan). SB-431542, a TGF-β inhibitor, was synthesized as described previously and was purchased from Wako (SB-431542*n*-Hydrate, Wako, Osaka, Japan) [20,21]. Tumors were measured twice weekly, and volume was calculated using the following formula: Volume = width^2^ × length × 0.52. Mice were randomly assigned to two treatment groups: control, n = 14 (20% DMSO/80% corn oil); SB-431542-treated, n = 15 (10 mg/kg body weight in 20% DMSO/80% corn oil, administered intraperitoneally three times per week starting one day after tumor cell inoculation [22]. Primary tumors were resected when the volume at day 10 post-injection of 4T1 cells. All mice were monitored daily and euthanized after 4 weeks. The metastases were dissected to snap-freeze for further analysis. All experimental procedures were conducted in accordance with the Japanese regulations on animal experiments and approved by the Institutional Animal Care and Use Committee of National Institute of Biomedical Innovation, Osaka, Japan.

### Peptide dimethyl labeling

Snap-frozen 4T1 metastatic lesions were weighed and washed twice with PBS. Protein extracts were prepared using the phase transfer surfactant (PTS) method as described previously [23]. Briefly, metastases samples were lysed with a 12 mM sodium deoxycholate, 12 mM sodium N-lauroylsarcosinate and 50 mM ammonium bicarbonate solution containing 1× PhosSTOP Phosphatase Inhibitor and Protease Cocktail (Roche Diagnostics, Indianapolis, IN, USA). Protein concentrations were measured using a DC protein assay kit (Bio-Rad, CA, USA), and 1 mg of each tumor sample was subjected to proteomic analysis. One mg protein lysates were reduced with 10 mM dithiothreitol, alkylated with 20 mM iodoacetamide, and digested with 1:100 (w/w) trypsin (Roche). The tryptic digest sample was desalted using C18 stage tips. [24]. An equal volume of ethyl acetate with 0.5% trifluoroacetic acid (TFA, Wako) was added. The mixture was vortexed for 1 min and centrifuged at 15,700 × *g* for 2 min, and the subaqueous phase was removed. Dimethyl labeling was carried out as previously described [25]. Briefly, the samples were labeled separately by incubation with CH_2_O and NaBH_3_CN (light), CD_2_O and NaBH_3_CN (medium) and ^13^CD_2_O and NaBD_3_CN (heavy) (Sigma-Aldrich Chemical Co, MO, USA) for 1 hr at room temperature. The labeling reaction was quenched by adding ammonium solution. The reaction was further acidified with 5% formic acid, then the differentially labeled samples were mixed and cleared using C18 stage tips.

### Separation with strong cation exchange chromatography (SCX)

The dimethyl-labeled peptides were fractionated using an HPLC system (Shimazu Prominence UFLC, Kyoto, Japan) fitted with an SCX column (50 mm × 2.1 mm, 5 μm, 300A, ZORBAX 300SCX; Agilent Technologies). The mobile phases consisted of Buffer A [25% acetonitrile and 10 mM KH_2_PO4 (pH3.0)] and Buffer B [25% acetonitrile, 10 mM KH_2_PO4 (pH 3.0), and 1 M KCl]. The labeled peptide was dissolved in 100 μl of Buffer A and separated at a flow rate of 200 μL/min using a four-step linear gradient; 0% B for 30 min, 0–10% B in 15 min, 10–25% B in 10 min, 25–40% B for 5 min, and 40–100% B in 5 min, and 100% B for 10 min. A total of 22 fractions were collected, vacuum-dried and desalted using C18 stage tips, and 2 μg of the digest was used for LC-MS/MS analysis [26].

### LC-MS/MS

Fractionated peptides were analyzed using an LTQ-Orbitrap Velos mass spectrometer (Thermo Fisher Scientific, Bremen, Germany) equipped with a nanoLC interface (AMR, Tokyo, Japan), a nanoHPLC system (Michrom Paradigm MS2) and an HTC-PAL autosampler (CTC, Analytics, Zwingen, witzerland). The analytical column was made in-house by packing Lcolumn2 C18 particles (Chemical Evaluation and Research Institute (CERI), Tokyo, Japan), into a self-pulled needle (200 mm length × 100 μm inner diameter). The mobile phases consisted of buffer A (0.1% formic acid and 2% acetonitrile) and B (0.1% formic acid and 90% acetonitrile). Samples dissolved in buffer A were loaded onto a trap column (0.3 × 5 mm, L-column ODS; CERI). The nanoLC gradient was delivered at 500 nL/min and consisted of a linear gradient of buffer B developed from 5% to 30% B in 180 min. A spray voltage of 2000 V was applied. Full MS scans were performed using an orbitrap mass analyzer (scan range m/z 350–1500, with 30 K fwhm resolution at m/z 400). The 10 most intense precursor ions were selected for the MS/MS scans, which were performed using collision-induced dissociation (CID) and higher energy collision-induced dissociation (HCD, 7500 fwhm resolution at m/z 400) for each precursor ion. The dynamic exclusion option was implemented with a repeat count of 1 and exclusion duration of 60 s. Automated gain control (AGC) was set to 1.00e + 06 for full MS, 1.00e + 04 for CID MS/MS, and 5.00e + 04 for HCD MS/MS. The normalized collision energy values were set to 35% for CID and 50% for HCD [27,28].

### Data acquisition with LTQ Orbitrap Velos

The MS/MS spectra acquisition of the dimethyl-labeled peptides was processed and quantified using Maxquant software (version 1.3.0.5) against the Uniprot.human.2011_11 database. The MS/MS search criteria were as followed; at least 1 unique peptide, the FDR of peptide identification set to 0.01 and minimum peptide length of 6 amino acids. The enzyme was trypsin, the dynamic modifications were set for oxidized Met (+16) and carbamidomethylation of cysteine was set as a static modification. MS/MS tolerance was set at 10 ppm. A dimethyl-based quantification method was chosen in Maxquant, with a mass precision requirement of 2 ppm for consecutive precursor measurements. Peptide ratios were then normalized against the median (log_2_).

### Western blotting

Whole tissue extracts were prepared in PTS buffer for proteomics analysis and were also used in western blots with three randomly selected mice per condition. Typically, 10–30 μg of total protein was loaded onto 4–20% Tris-glycine SDS PAGE gels (DRC, Tokyo, Japan) and transferred to PVDF membranes (Millipore, Temecula, CA). Membranes were probed for the following antibodies: anti-vimentin (1:3000, D21H3, #5741, Cell Signaling Technology Japan, Tokyo, Japan), anti-Eno-1 (1:1000, AB1, AV34376, Sigma-Aldrich), anti-HSP90a/b (1:1000, F-8, sc-13119, Santa Cruz Biotechnology, California, USA), anti-eIF4G1 (1:1000, #2858, Cell Signaling Technology Japan), anti-eIF4E (1:1000, #9742, Cell Signaling Technology Japan), and anti-eEF2 (1:1000, #2332, Cell Signaling Technology Japan). Anti-β-actin (1:5000, AC-74, A-2228, Sigma-Aldrich) was used to assess equivalence of protein loading. Peroxidase-conjugated secondary antibodies were used at 1:5000 dilutions, and signals were detected by ECL (Thermo Scientific Pierce, Rockford, IL).

### Immunohistochemistry

Formalin-fixed, paraffin-embedded tissue was immunostained with antibodies against anti-phospho-histone H3 (1:100, Ser10, #9701, Cell Signaling Technology Japan) and anti-vimentin (1:300), anti-eIF4G1 (1:100), anti-eIF4E (1:100) and anti-Eno-1 (1:100) antibodies. The sections were subsequently incubated with Dako Envision anti-rabbit secondary antibody (Dako, Tokyo, Japan) and visualized with liquid diaminobenzidine (DAB, Dako). Nuclei were counterstained with Mayer’s hematoxylin solution (Wako Pure Chemical Industries Ltd., Osaka, Japan), and images were taken on a Zeiss LSM710 confocal laser microscope (Zeiss, Jena, Germany). Nuclear phosphorylated-histone H3 staining was quantified for individual metastatic nodules. The area of positive staining was semiquantitatively assessed with Image Pro’s Count/Size and Smart tool in at least three high-power fields using Image Pro Premier software (Media Cybernetics, Tokyo, Japan) at 100× magnification. OCT compound-embedded tissues were cut to 5 μm thickness using a cryostat (HYRAX C50, Zeiss). Cryosections were fixed with cold 100% acetone for 5 min, blocked with 5% milk, and then stained with anti-vimentin antibody with Alexa Fluor 488 conjugated anti-rabbit IgG (Invitrogen) and Alexa Fluor 488 anti-mouse CD31 (102414, Biolegend, CA, USA). Sections were counterstained with DAPI. At least three randomly selected high power fields (200×) were assessed for each metastasis within a given lung section using a Zeiss LSM710 confocal laser microscope (Zeiss, Jena, Germany), and quantitated using LSM710/780 ZEN software (Zeiss).

### Statistical analysis

Statistical analysis was performed using Prism version 6.0 (GraphPad software Inc., San Diego, CA). Student’s *t*-test and the Mann-Whitney *U*-test were used to compare differences between the two groups. A p value of less than 0.05 was considered to indicate a significant difference, p values: * <0.05, ** <0.001. The LC-MS/MS data were exported into SIMCA 13 software for analysis and visualization by multivariate statistical methods previously described [29]. Principal component analysis (PCA) auto-fit was computed on four samples from each group against a mixture reference. Pathway mapping and network visualization were assisted by Ingenuity Pathway anlysis (IPA) (www.ingenuity.com).

## Results

### The TGF-β small molecular antagonist, SB-431542, inhibits 4T1 xenograft tumor metastasis

**Fig 1:**
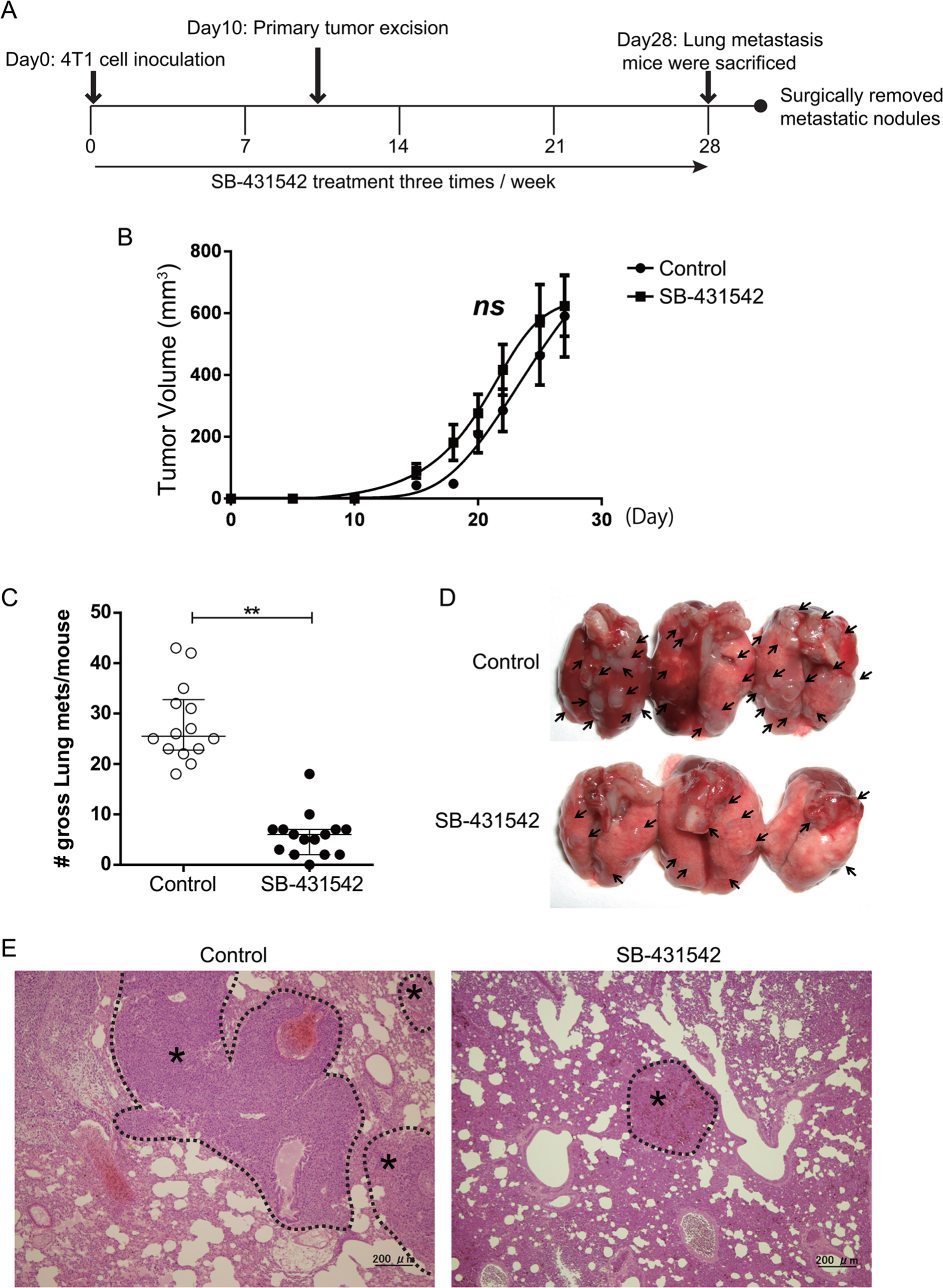
Orthotopic metastasis model demonstrates that a specific inhibitor of TGF-β receptor kinase, SB-431542, decreases lung metastasis but does not significantly alter growth of the primary tumor. (A) Scheme for the experimental approach using 4T1 metastasis model. 4T1 tumor cells (1×10^4^ cells) were transplanted into the mammary fat pad of Balb/c mice. Mice bearing 4T1 mammary tumors were treated three times weekly with SB-431542 (10 mg/kg body weight) or vehicle (20% DMSO/80% corn oil). At day 10 post-injection of 4T1 cells, primary tumors were surgically excised, and the mice were kept alive to allow the tumor to metastasize to the lung. (B) Graph showing relative primary tumor growth of 4T1 cells over time. Data are presented as mean ± SEM. (ns = not significant, control, n = 14; SB-431542, n = 15, unpaired *t*-test). (C) The number of gross metastasis (control, n = 14; SB-431542-treated, n = 15) was counted. The administration of SB-431542 markedly reduced both the number and the size of the metastasized tumor compared to the control group. Lungs were collected after 40 days and the lung surface was examined for the metastasis. The number of visible lung metastasis was counted. ** p<0.001.; Mann Whitney *U*-test. Data are presented as the median ± SEM. (D) Representative gross lung images from control and SB-431542-treated groups are shown from control (top) and SB-431542-treated (bottom) animals, with metastases visible at the lung surface marked by bold black arrows. (E) Representative histological view of lung metastases treated with vehicle, DMSO (left) and SB-431542 (right). H&E staining; black dotted lines demarcate tumor parenchyma (*) from the normal lung tissue.

The 4T1 cell line is a highly metastatic murine breast cancer with a proclivity for lung metastasis. SB-431542 treatment was begun at the time of inoculation. Ten thousand 4T1 cells were implanted into the mammary fat pad, and the mice received intra-peritoneal (i.p.) administration of SB-431542 (10 mg/kg body weight in 20% DMSO/80% corn oil) or vehicle (20% DMSO/80% corn oil) as control three times a week (Fig 1A). The treatment did not affect the growth of the primary tumor (Fig 1B). A large number of metastasized tumor masses were observed at day 28 in the lung. As seen in other TGF-β antagonist studies [14,30], the administration of SB-431542 markedly reduced both the number and size of metastasized tumors compared with the control group at metastatic sites (Fig 1C and 1D). The reduction in the size of microscopic nodules was confirmed in lung metastases by pathological examination (Fig 1E). These results clearly showed that the small-molecule TGF-β antagonist SB-431542 can suppress metastasis of 4T1 cells to the lungs from the orthotopic site and that this effect is not secondary to effects on the primary tumor.

**Fig 2:**
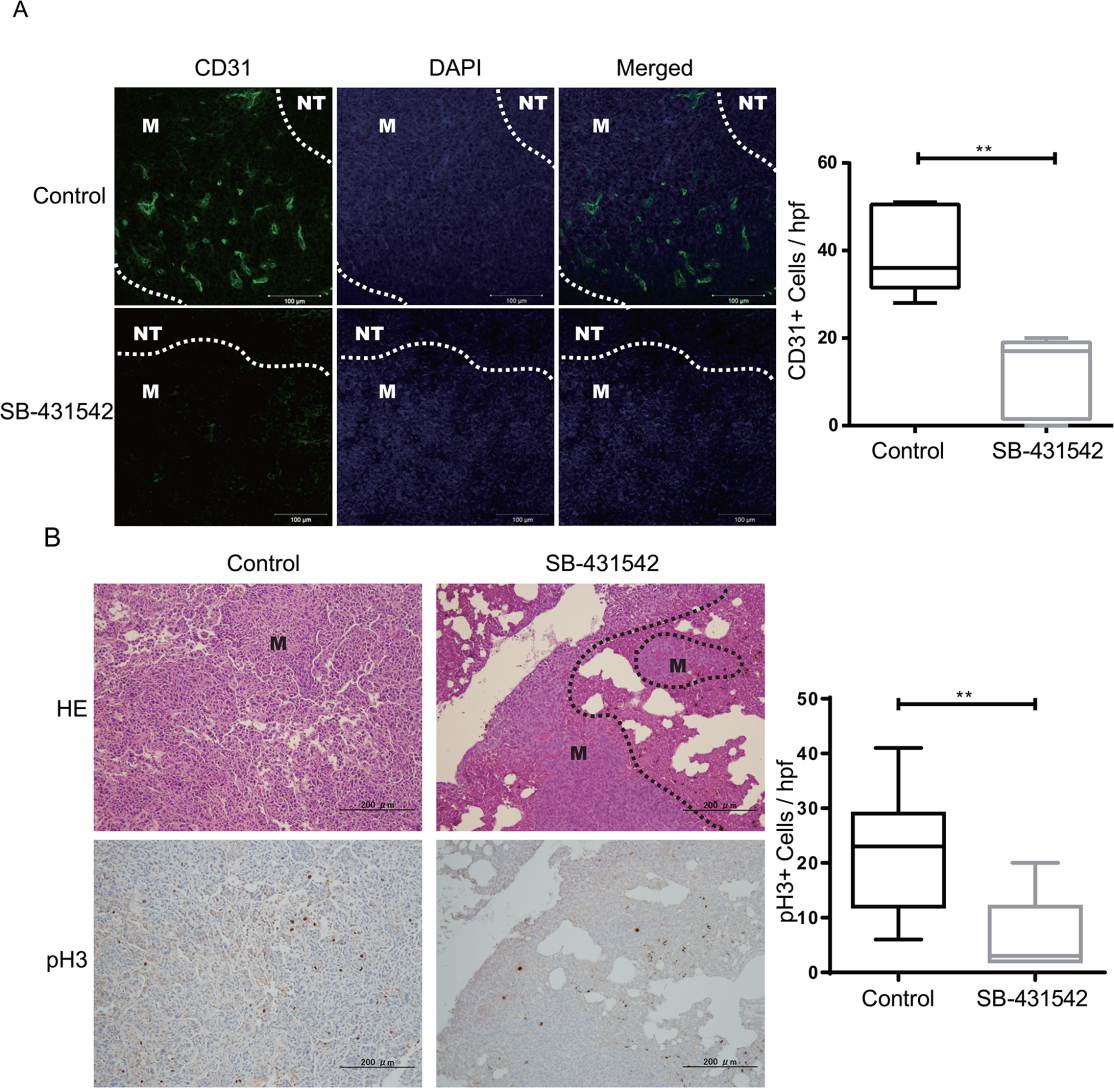
SB-431542 treatment inhibits the angiogenesis and proliferation of metastasized tumors. (A) Representative images of metastasized tumors with CD31 (green) and DAPI (blue) staining per vessel density. Scale bars represent 100 μm. White dotted lines demarcate metastases (M) and normal lung tissue (NT). (B) CD31 immunostaining was captured using a Zeiss microscope, and the number of positive cells was counted. The Box blot represents the number of positive cells/field. The results are shown as the upper and lower quartiles and the mean, and the whiskers show the maximum and minimum values; there are 3 tumors/group. Black dotted lines demarcate metastases (M) and normal lung tissue. (C) Paraffin-fixed sections were assessed by H&E staining (top panel) and were stained for proliferation using phospho-Histone H3 (pH3) antibody (bottom panel). (D) The number of pH3-positive cells was counted and plotted as shown to obtain a statistical value. The mean number of pH3-positive cells ± SEM for 3 tumors/group is shown, ** p<.0.001 with unpaired *t*-test; hpf, high power field.

To investigate the possible underlying pathological phenotypes in this model, we assessed proliferation, apoptosis and vessel density in the tumor samples using anti-phospho-histone H3 (pH3), cleaved caspase-3 (CC3) and CD31 antibodies, respectively. Quantification of pH3 and CD31 staining revealed a decrease in cell proliferation and vasculature area in the parenchyma cells of the metastases in SB-431542-treated groups (Fig 2A and 2B). As expected, SB-431542 treatment resulted in statistically significant pathological differences in the microvascular density of the metastasized tumors (p = 0.0079) and in cell proliferation (p = 0.0006) compared to the control group using the Mann-Whitney *U*-test. Although no significant difference was seen in apoptosis (p = 0.4, not significant, S1 Fig), these results are consistent with those of other studies suggesting that the blockade of the TGF-β receptor effectively inhibits metastasis by triggering anti-tumor angiogenesis and growth arrest [14].

### Protein profile using quantitative proteomic analysis with dimethyl labeling

**Fig 3:**
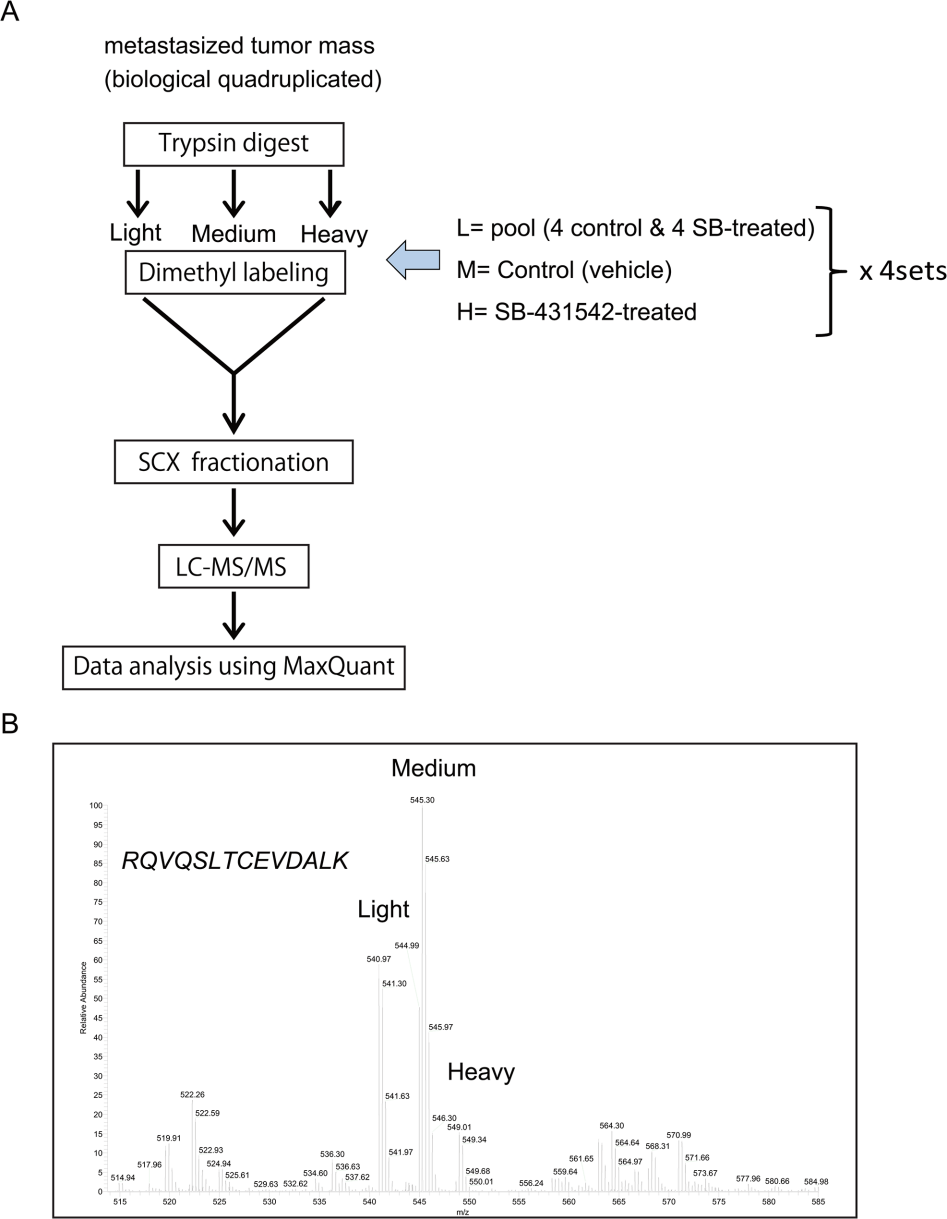
Protein expression profiling of 4T1 metastases tumor using dimethyl labeling with triplex stable isotopes. (A) Schematic representation of the proteomics approach. Lung-metastasized 4T1 tumor samples were isolated from each group (control and SB-431542-treated tumors). Subsequently, the tumor protein was lysed, and differential protein expression was detected by relative quantification using dimethyl-labeling (light, medium and high) followed by an SCX-LC-MS/MS (LTQ Orbitrap Velos) analysis for each set of sample quadruplicates. Isotope-light was used as the reference sample and contained mixed aliquots of all control and SB-431542-treated tumor samples. Four sets of dimethyl-labeled experiments were performed to compare the protein profiles of 8 metastasized tumor samples. (B) Representative dimethyl labeled-based LC-MS/MS spectrum for one of the peptides from vimentin (P20152) showing RQVQSLTCEVDALK, a double charged peptide.

To identify any factors involved in regulating TGF-β activation in metastasis at the protein level, the metastases were macro-dissected from the normal lung tissue in control and SB-431542-treated groups, and tumor proteins were analyzed using mass spectrometry (Fig 3A). We pooled metastases from an individual mouse of randomly selected four control and four SB-431542-treated metastases samples, and a mixture of all eight samples was used as a reference. First, each sample was lysed using the PTS method and digested with trypsin. The reference-pooled peptides, control, and SB-431542-treated peptides were dimethyl-labeled with stable isotope tags; “light” (L), “medium” (M) and “heavy” (H), respectively. Labeled peptides were mixed and separated into 22 fractions by cation exchange chromatography. We measured each fraction by nanoLC coupled to high-resolution quantitative mass spectrometric analysis using the LTQ Orbitrap Velos. Raw data from LC-MS/MS was analyzed using Maxquant (ver. 1.3.1.5) to identify and quantify peptides and protein. The criteria of protein thresholds were set to a false discovery rate (FDR) of 0.01 and at least 1 identified unique peptide, and 36,239 peptides from 6,694 proteins were identified. Maxquant determines the peptide ratios of M/L and H/L that indicate the relative abundance of proteins in control and SB-431542-treated samples with respect to the reference mixture, and 4,531 proteins were quantitatively identified between the two groups. A representative LC-MS/MS spectrum of one of the dimethyl labeled peptides of vimentin is shown in Fig 3B.

**Fig 4:**
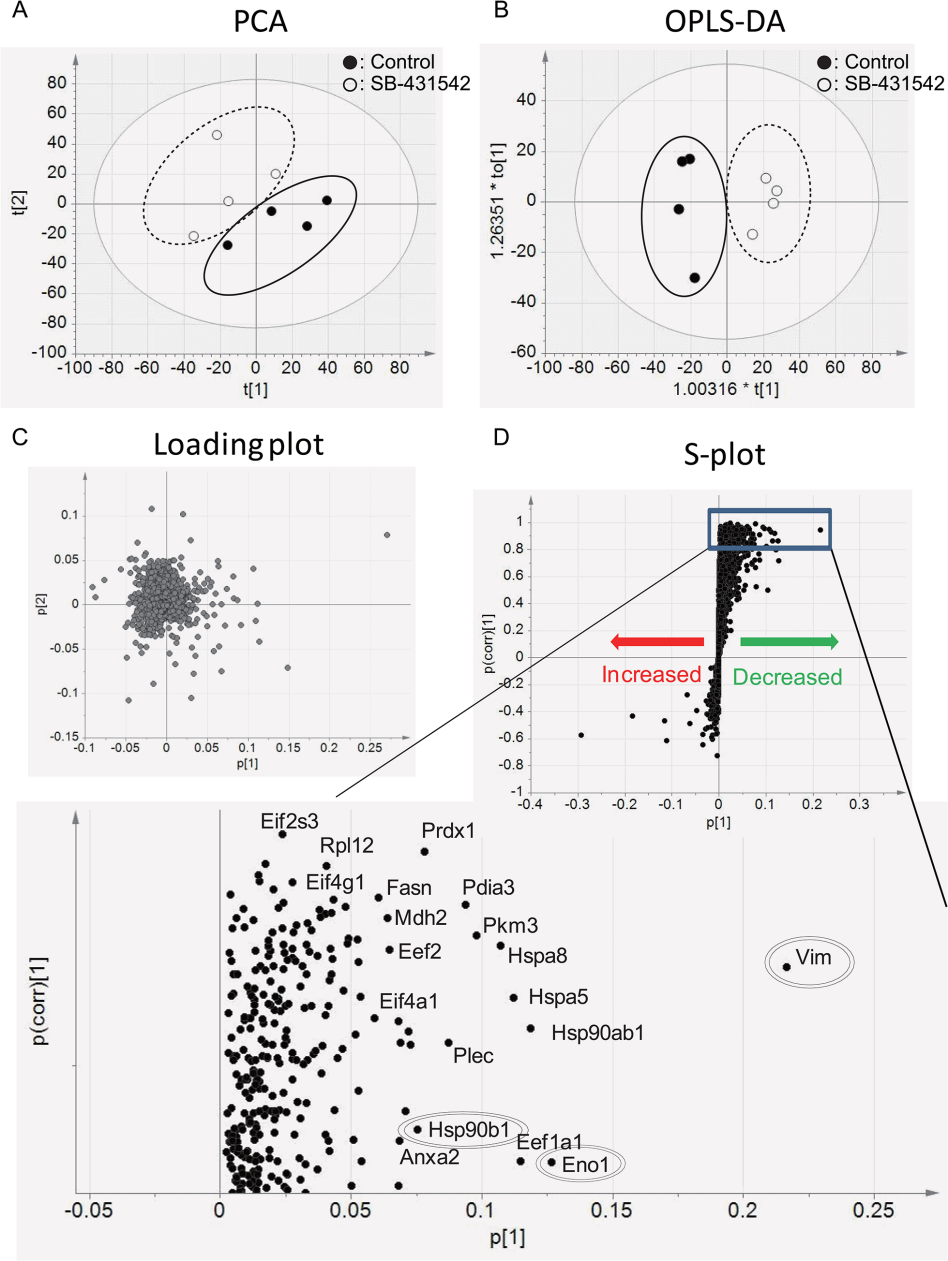
Protein profiling between metastasized tumors of control and SB-431542-treated groups. (A) A PCA plot based on the peptide profile in control and SB-431542-treated tumors using SMCA13 for the multivariate analysis and (B) corresponding loading plot. (C) OPLS-DA plot discriminates proteins from those two groups. Black dots represent the control group (n = 4) and white dots represent the SB-431542-treated group (n = 4). (D) S-plot shows the significance of protein variations between control and SB-431542-treated groups. Each black dot represents individual proteins. Black dots in the positive directions indicate the decreased proteins in SB-431542-treated tumors. The increased proteins in SB-431542-treated tumors are presented as dots in the negative direction. Vimentin (Vim), Hsp90ab1 and Eno1 were double-line-circled as the top proteins of the highest confidence and greatest contribution separation between the untreated and SB-431542-treated mice. (S1 Table). A magnified picture of the significantly reduced proteins in SB-431542-treated tumors is shown (p(corr) > 0.80).

We further analyzed the data using SIMCA13 software to study proteomic profiling of metastasis-associated proteins following TGF-β blockade in four metastases. After data normalization, LC-MS/MS coupled with Principal component analysis (PCA) showed a difference in the peptide profile between control and SB-431542-treated groups (Fig 4A). Subsequently, based on protein data, orthogonal projections to latent structures-discriminant analysis (OPLS-DA) indicated an apparent separation between the groups (Fig 4B). To identify the main proteins responsible for the separation between the groups, their loading plot with combined covariance and correlation coefficients were displayed with using sigmoid-plot (S-plot, Fig 4C and 4D). Values of correlation coefficient (p(corr)) between medians of quadruplicates of experiments above 0.80 indicate good reproducibility [29]. The loading was shown to be significant, discriminating 431 proteins for the clustering patterns. Those positive signals indicated the presence of down-regulated proteins in the SB-431542-treated samples in comparison with controls, including a known TGF-β-targeted protein, vimentin. However, the signals in the negative direction indicated the up-regulated proteins in SB-431542-treated samples that included hematogenous proteins, which may reflect contamination during the sample preparation, and none of up-regulated proteins showed statistically confident values in this model (p <0.85). Therefore, we focused on the down-regulated proteins in SB-431542-treated samples. The list of the significantly differentially expressed proteins was summarized for a correlation efficient (p(corr) <0.80. (S1 Table).

### Identification of eIF proteins as TGF-β targets in 4T1 lung metastases

**Fig 5:**
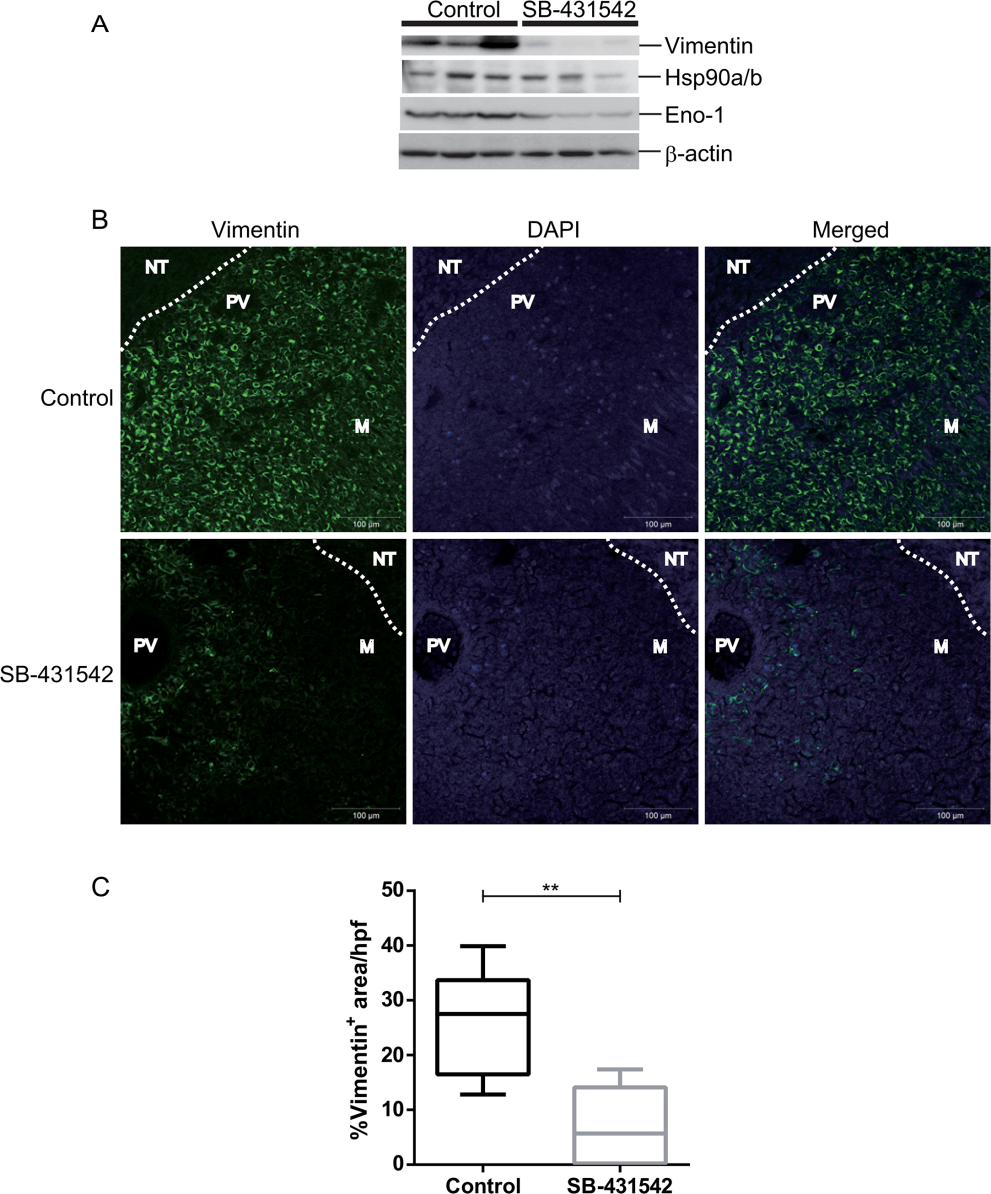
Validation of protein expression using western blot and immunohistochemistry. (A) Metastases lysates were prepared from three mice per group and evaluated by western blot with anti-vimentin, anti-Hsp90a/b, and anti-Eno1 antibodies. β-actin was used as internal normalization. (B) Representative image of positive vimentin staining is shown exclusively in the metastases lesion and not in the surrounding lung tissue. White dotted lines represent a boundary of tumor and surrounding normal lung tissue. M, metastases; NT, normal lung tissue; PV, pulmonary vein. (C) Randomly selected high power fields were immunostained with vimentin and were quantitated using Zeiss software with the % area occupied by metastasized nodules in 4T1 metastasized tumors. A significant reduction in the number of vimentin positive cells was seen in the SB-431542-treated tumors. Scale bars represent 100 μm; hpf, high power fields.

To validate the relative expression levels of proteins in the metastases of 4T1 model with and without treatment, we conducted western blotting and immunohistochemistry assays. Using western blotting, the TGF-β target proteins of interest, namely, vimentin, Eno-1 and Hsp90a/b1, which were at the top of the list as the highest confidence and greatest contribution separation between the two groups (Fig 4D and S1 Table), were confirmed to be down-regulated in SB-431542-treated tumors compared to the control (Fig 5A). The amount of sample loading was normalized based on Sypro ruby protein staining (not shown) and β-actin levels. We performed immunohistochemistry to examine the localization of vimentin and Eno-1 protein expression (Fig 5B and S2 Fig). Both vimentin and Eno-1 immunostaining showed that the proteins were mainly expressed in metastases, and the number of vimentin-positive cells was quantified and found to be significantly reduced in the SB-431542-treated tumors (Fig 5C).

**Fig 6:**
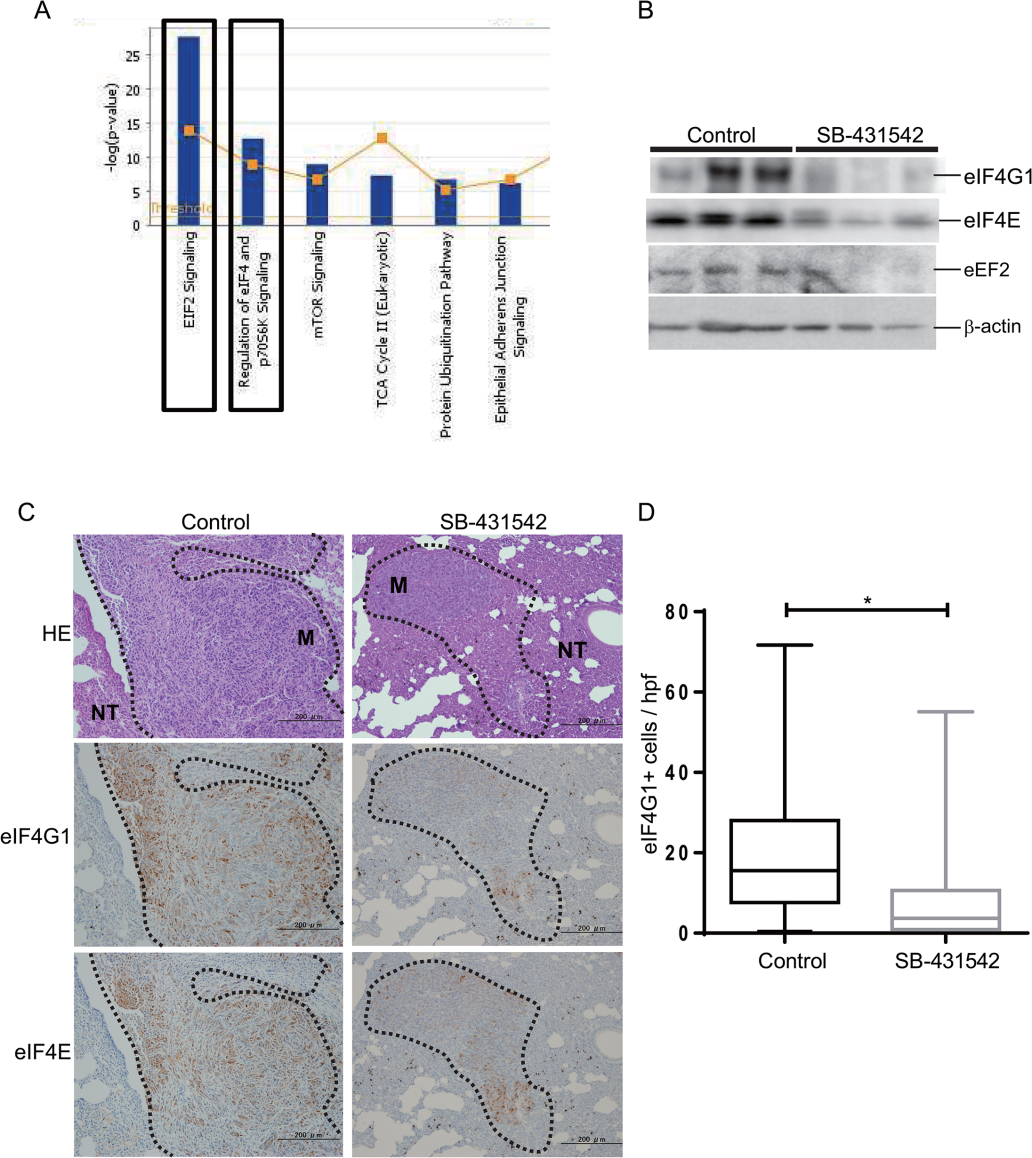
EIF signaling was the most enriched canonical IPA pathway of the down-regulated proteins in SB-431542-treated groups. (A) Pathway analysis conducted using IPA (www.ingenuitypathway.com) showed a ranking of the most enriched pathways from the down-regulated proteins in SB-431542-treated groups. (B) Whole cell lysates from each group were evaluated by western blot with the EIF family of proteins: anti-eIF4G1, anti-eIF4E, and anti-Eef2 antibodies. (n = 3/group).β -actin was used as a loading control. (C) Sections were assessed by H&E staining (top panel), and immunohistochemistry was performed using anti-eIF4G1 (middle panel) and anti-eIF4E (bottom panel) antibodies. Both eIF4G1 and eIF4E immunostaining in 4T1 lung metastasis showed the heterogeneity of staining patterns seen among individual metastases. 100× magnification. Tumor sections are boxed with a black dotted line, M, metastases; NT, normal lung tissue. (D) eIF4G1- and eIF4E-positive areas were semiquantified using Image Pro Premier Software in three randomly selected high power fields from each samples. Data are represented as box plots (n = 3/group), ** p<0.001 using an unpaired *t*-test.

The differences in the protein profile of lung metastases treated with the TGF-β antagonist might identify novel metastasis-associated proteins and pathways that are responsive to TGF-β signals. To identify pathways and active networks in the metastatic lesions, the down-regulated proteins in SB-431542-treated groups were analyzed using Ingenuity Pathway Analysis (IPA) software. The signaling pathways involving the eukaryotic translation initiation factors eIF2 and eIF4 were the most enriched pathways among other known tumor-related pathways, such as the protein ubiquitination and TCA cycle II pathways (Fig 6A and S3 Fig). Interestingly, both the eIF2 and eIF4 signaling pathways have recently been implicated in tumor progression signatures in several cancers, including breast, colon and liver [31–33]. The eIF family proteins are also key regulators of tumor angiogenesis that function by upregulating angiogenic factors such as VEGFA and HIF1α [34]. We further evaluated the expression levels of eIF-related proteins using antibodies. eIF2α (data not shown), eIF4G1, eIF4E and eukaryotic elongation factor eEF2 protein expression was decreased in SB-431542-treated lung metastases compared to untreated metastases, in agreement with the results of the proteomic analysis (Fig 6B). We also observed the localization of eIF4G and eIF4E in paraffin-embedded metastases using immunohistochemistry, and the expression of these two proteins following SB-431542 treatment was lower in parenchyma cells compared to the control (Fig 6C). Importantly, we noted only eIF4G1 expression was significantly decreased in SB-431542-treated group, but not the other eIF family members, at the mRNA level, which means that this overall effect of TGF-β antagonism on eIF signaling pathway would not have been identified by transcriptomic approaches. In contrast, mRNA of the known TGF-β target *Serpine1*, showed a clear reduction in SB-431542-treated tumors (S4 Fig). From our observations, we speculate that SB-431542 effectively inhibited 4T1 tumor metastasis at least in part by blocking TGF-β -driven enhancement of eIF translational activity.

## Discussion

In this study, we evaluated protein expression profiles in a murine 4T1 breast cancer metastasis model using a quantitative proteomic approach and multivariate statistical analysis. The TGF-β signaling antagonist SB-431542 significantly inhibits lung metastasis in association with reduced angiogenesis and cell proliferation. Our proteomic analysis demonstrated that SB-431542 significantly inhibited the expression of a number of individual proteins associated with macroscopic tumor formation in the lung. The top proteins on the list included vimentin, which is known to have a crucial role in epithelial transformation during invasion and metastasis [35]; Eno-1, which has functions in tumor growth, differentiation, colony formation, and survival [36]; and of HSP-90a/b1 which functions in survival, migration and as a negative regulator involved in proteasomal ubiquitin-dependent protein catabolic processes [36]. Interestingly, the level of vimentin mRNA was unchanged on treatment, showing that this approach can pick up key molecular changes that would not be detected by transcriptomic approaches.

In looking for pathway and functional enrichment among the set of differentially expressed proteins, we found that EIF signaling was the most enriched pathway, and several eIF family proteins were down-regulated in lung metastases in this model following treatment with SB-431542. Eukaryotic mRNA translation is regulated by recruitment of the eukaryotic initiation factors eIF4F complex, which is composed of three proteins: the cap-binding protein eIF4E, scaffolding protein eIF4G and ATP-dependent RNA helicase eIF4A [34,37,38]. Interestingly, many onco-proteins have long highly structured 5’UTRs that makes them very dependent on the level of activity of the eIF complex for efficient translation [39]. All of these three proteins were differentially expressed in this model, but were unaffected at the mRNA level (S4 Fig), suggesting that TGF-β specifically upregulates eIF family protein expression in lung metastasis. Overexpression of eIF family member genes has previously been shown to be associated with metastatic progression and reduced patient survival [40,41]. Among the eIF proteins, we found that eIF4G1 was one of the most differentially expressed proteins lung metastases, when comparing the control and SB-431542 treatment groups, and its expression was exclusively localized to the tumor and not the surrounding lung tissue. Interestingly, it has been reported that high levels of eIF4G1 expression selectively increase translation of mRNAs involved in cell growth, proliferation and bioenergetics, while only partially reducing overall protein synthesis [42]. Elevated eIF4G1 was shown to drive the formation of tumor cell emboli to promote invasion in inflammatory breast cancer through a unique translation process that is different from that of other eIF proteins and was proposed to involve enhanced proliferation, prevention of autophagy and release from nutrient control [32]. Consistent with this study, we showed high level expression of the eIF4G1 protein in the untreated 4T1 lung metastases. Moreover, eIF4G1 was previously shown to be significantly overexpressed in human inflammatory-type breast cancer, which exhibits a similar pathological phenotype to that of the 4T1 tumor, in which 40–50% of cells involved in the overall tumor mass are immune cells [43]. Importantly, in this study we have shown for the first time that the eIF4 protein complex is upregulated by TGF-β or closely-related TGF-β superfamily members in metastatic lesions. TGF-β has previously been shown to stimulate translation in other biological settings, but the reported mechanisms involved TGF-β regulated activation of the Akt-mTOR pathway, leading to phosphorylation of S6 kinase and eIF4E-BP1 [44] or activation of PI3K and Mnk1 kinases leading to phosphorylation of eIF4E [45]. Thus here we have identified a novel mechanism of TGF-β regulation of translation involving upregulation of the protein components of the eIF4 complex rather than their post-translational modification. Our observation of the loss of eIF4G1 expression upon SB-431542 treatment is interesting enough to pursue further, as the Badura *et al*. study showed that eIF4G1 expression was associated with drug resistance [42]. Thus, the eIF complex may act as a potent mediator of the cancer-promoting effect of TGF-β signals.

One caveat in the interpretation of our study is that in addition to inhibiting the TGF-β type I receptor kinase, Alk5, SB-431542 also inhibits the Alk4 and Alk7 kinases that mediate signaling downstream of the closely-related TGF-β superfamily members, the activins, certain GDFs and nodal [21,46]. Thus currently we cannot unambiguously attribute the proteomic changes we observe to inhibition of TGF-β signaling as opposed to inhibition of these closely related family members. The related superfamily members are less well-studied than TGF-β in the context of tumorigenesis, but an emerging literature suggests that they can have similar pro-oncogenic effects to the TGF-βs, with many overlapping molecular targets [6]. It will be important in future studies to dissect out the relative contributions of the different TGF-β family members to the metastatic process, but nevertheless, our study clearly indicates that targeting this branch of the TGF-β superfamily has therapeutic efficacy associated with major changes in a novel downstream effector pathway, the eIF pathway. Another potential limitation of our approach is that our SB-431542 treated metastases had to have had some resistance to the effect of therapy in order to have formed at all. However the treated metastases were significantly smaller than the untreated metastases, so we are likely identifying pathways that are relevant to the efficiency of metastatic outgrowth and colonization rather than early survival and seeding.

Inhibitors of the TGF-β signaling pathway, such as small-molecule receptor kinase inhibitors and ligand-neutralizing monoclonal antibodies, have been developed by pharmaceutical companies [7]. Some antagonists reached phase III clinical trials and have been beneficial to many cancer patients. However, TGF-β action is highly context-dependent and influenced differently by cell type, interactions with non-canonical pathways, culture conditions and disease stage *in vivo*, reflecting the complex nature of TGF-β biology. Conceptually, the ideal therapeutic strategies would target the pro-oncogenic activity of TGF-β exclusively without side effects while avoiding effects on TGF-β tumor suppression. Such selectivity could come from a variety of different sources. In one scenario, targeting the central TGF-β signaling pathway at different levels or to different degrees might inherently discriminate between the desirable and undesirable effects of TGF-β. In an alternative scenario, identification of the downstream mediators of the pro-oncogenic effects of TGF-β opens up the possibility of directly targeting these effectors.

In relation to the first scenario, previous preclinical studies showed that SB-431542 does not completely block the growth inhibitory effect of TGF-β in normal epithelial cells, but efficiently inhibits TGF-β -induced proliferation of tumor cells, blocks autocrine TGF-β signaling, and inhibits TGF-β -induced EMT and invasiveness, suggesting there may be some selectivity for pro-oncogenic effects of TGF-β [20,47,48]. In our present study, SB-431542 resulted in regression of the lung metastasis but did not alter growth of primary tumor in the 4T1 model, whereas a monoclonal antibody that neutralizes the three TGF-β ligands decreased both primary tumor growth and lung metastases in the same model, though the effect on metastases was less profound than was seen here with SB-431542 [14]. Thus targeting different levels on the TGF-β signaling pathway may lead to different outcomes in complex cancer models. However since, as noted above, SB-431542 also inhibits signaling downstream of closely related TGF-β superfamily members, some of the differences between the two approaches may relate to the expanded specificity of the kinase inhibitor. This is clearly an area that needs further investigation. Indeed, it would be interesting to combine TGF-β ligand neutralizing antibodies and small molecule receptor kinase inhibitors to address whether such an approach could increase overall therapeutic efficacy. In relation to the second scenario for selective blockade of pro-oncogenic effects of TGF-β, our proteomics approach has identified potential novel downstream mediators of the effector arm of the TGF-β-driven pro-metastatic program that could serve as new targets for therapy. Such an approach would avoid the potential for interfering with the tumor-suppressive and homeostatic effects of TGF-β, an issue that is a major concern when inhibiting the upstream signaling events.

Here we focused on identifying key underlying mechanism of TGF-β regulation of metastasis *in vivo* by applying global proteomic analysis to metastatic lesions isolated from a preclinical mouse model of breast cancer metastasis treated with a TGF-β pathway inhibitor. The global proteomic analysis demonstrated significant differential expression of proteins between control tumors compared to SB-431542-treated tumors in lung metastasis, and represents an exciting new approach to identify critical factors and signals associated with tumor metastasis. Importantly we identified novel TGF-β targets that are unchanged at the mRNA level and so would have been missed by transcriptomic approaches. Our study suggests that the TGF-β signal upregulates eIF proteins to initiate mRNA translation for downstream signaling events that promote pathological phenotypes, such as tumor cell proliferation and angiogenesis. Many but not all of the overexpressed proteins were exclusively localized in metastatic lesions compared to the surrounding lung tissue, suggesting that TGF-β signal blockade by SB-431542 significantly alters the proteome of lung metastases in association with reduced local angiogenesis and tumor cell proliferation at the metastatic site.

## Conclusions

From our data, we propose that the blockade of TGF-β receptor effectively inhibits metastasis by reducing tumor cell protein translation through reduced eIF signaling, and consequently suppressing angiogenesis and tumor cell proliferation in metastases. To our knowledge, this is the first study showing a link between TGF-β and expression of components of the eIF family members in breast cancer metastasis, thus demonstrating the power of this type of quantitative proteome analysis. Furthermore, our result underscores the potential of eIF signaling as a therapeutic target for breast cancer metastasis and suggests new mechanisms whereby TGF-β may promote the later stages of cancer progression.

## Supporting Information

S1 Fig No significant differences were observed in apoptosis between control and SB-431542-treated tumors in lung metastasis. Representative immunostaining of cleaved caspase-3 (CC3) expression in lung metastases nodules in control and SB-treated tumors. 200× magnifications, green: CC3; blue: DAPI. Cleaved caspase-3 staining in tumor cells was quantified, and values are plotted in the box plot. No significant difference was observed in CC3 staining between the control and SB-431542 treated samples.
(EPS)

S2 Fig Eno-1 expressed heterogeneously in the tumor parenchyma of lung metastasis. Corresponding H&E staining (top panel) for tumor sections of Eno-1 immunostaining in control (bottom left) and SB-431542-treated (bottom right) tumors. 100× magnification; scale bar, 200 μm. H&E staining; black dotted lines demarcate tumor parenchyma (*) from the normal lung tissue.
(EPS)

S3 Fig Ingenuity Pathway Analysis shows involvement of a novel signaling pathway in TGF-β. eIF2, eIF4 and p70S6K signaling pathways are generated by IPA. eIF2 and eIF4 signaling pathways were the top two pathways in the top 10 functions/diseases associated with the SB-431542-treated groups. Each node represents a protein; proteins in pink-shaded nodes were found in the proteome analysis, while proteins in clear nodes were not found in this study.
(EPS)

S4 Fig Loss of statistical power in mRNA expression for the proteins of interest. RNA was extracted using QIAshredder and an RNeasy plus Mini kit (Qiagen, Alameda, CA), and qPCR was performed using cDNA generated from 100 ng total RNA with a 1^st^ Strand cDNA synthesis kit and random oligonucleotides (Roche). Primers were designed using the mouse qPrimerDepot program (http://mouseprimerdepot.nci.nih.gov/). Primer sequences are listed in S2 Table. Quantitative PCR reactions were carried out in triplicate using Kapa SYBR Fast (KAPA Biosystems, Boston, MA) with an ABI Prism 7500 System. Values were quantified using the comparative CT method, and samples were normalized to 18S ribosomal RNA. Relative mRNA level of Serpine1, Vimentin and eIF family members, eEF2, eIF4g1, eIF4E, eEF1A1, eIF2S3 and eIF4A1 was evaluated quantitatively by RTq-PCR. 18S mRNA was used as a normalization control for all samples. The ratio of expression in control and SB-431542-treated tumors was determined for each of the tumor samples. n = 3/group. Values are the mean ± standard deviation with error bars.
(EPS)

S1 Table List of significant differentially expressed proteins in the lung metastases between the untreated *vs* SB-431542-treated groups. Lung metastases samples from untreated and treated with SB-431542, were subjected to iTRAQ labeling and LC-MS/MS analysis to profile their expression levels. The list of protein ranking, based on the OPLS-DA (Fig 4B), indicates the highest confidence and greatest contribution separation between the untreated and SB-431542-treated mice, p[1]>0.02. Their expression ratios, control/ SB-431542, represent a significant downward trend in mean value ((p(corr)> 0.80, n = 4 per group).
(DOCX)

S2 Table PCR primer sequences used in this study. (DOCX)

## References

[1] Sotiriou C, Piccart MJ (2007) Taking gene-expression profiling to the clinic: when will molecular signatures become relevant to patient care? Nat Rev Cancer 7: 545–553. 17585334 10.1038/nrc2173

[2] Weigelt B, Peterse JL, van 't Veer LJ (2005) Breast cancer metastasis: markers and models. Nat Rev Cancer 5: 591–602. 16056258 10.1038/nrc1670

[3] O'Shaughnessy J (2005) Extending survival with chemotherapy in metastatic breast cancer. Oncologist 10 Suppl 3: 20–29. 16368868 10.1634/theoncologist.10-90003-20

[4] Massague J (2008) TGFbeta in Cancer. Cell 134: 215–230. 10.1016/j.cell.2008.07.001 18662538 PMC3512574

[5] Roberts AB, Wakefield LM (2003) The two faces of transforming growth factor beta in carcinogenesis. Proc Natl Acad Sci U S A 100: 8621–8623. 12861075 10.1073/pnas.1633291100 PMC166359

[6] Wakefield LM, Hill CS (2013) Beyond TGFbeta: roles of other TGFbeta superfamily members in cancer. Nat Rev Cancer 13: 328–341. 10.1038/nrc3500 23612460 PMC7608560

[7] Akhurst RJ, Hata A (2012) Targeting the TGFbeta signalling pathway in disease. Nat Rev Drug Discov 11: 790–811. 10.1038/nrd3810 23000686 PMC3520610

[8] Bonafoux D, Lee WC (2009) Strategies for TGF-beta modulation: a review of recent patents. Expert Opin Ther Pat 19: 1759–1769. 10.1517/13543770903397400 19939191

[9] Yingling JM, Blanchard KL, Sawyer JS (2004) Development of TGF-beta signalling inhibitors for cancer therapy. Nat Rev Drug Discov 3: 1011–1022. 15573100 10.1038/nrd1580

[10] Bao L, Haque A, Jackson K, Hazari S, Moroz K, Jetly R, et al (2011) Increased expression of P-glycoprotein is associated with doxorubicin chemoresistance in the metastatic 4T1 breast cancer model. Am J Pathol 178: 838–852. 10.1016/j.ajpath.2010.10.029 21281816 PMC3069823

[11] Tao K, Fang M, Alroy J, Sahagian GG (2008) Imagable 4T1 model for the study of late stage breast cancer. BMC Cancer 8: 228 10.1186/1471-2407-8-228 18691423 PMC2529338

[12] Issa A, Gill JW, Heideman MR, Sahin O, Wiemann S, Dey JH, et al (2013) Combinatorial targeting of FGF and ErbB receptors blocks growth and metastatic spread of breast cancer models. Breast Cancer Res 15: R8 10.1186/bcr3379 23343422 PMC3672810

[13] Nam JS, Suchar AM, Kang MJ, Stuelten CH, Tang B, Michalowska AM, et al (2006) Bone sialoprotein mediates the tumor cell-targeted prometastatic activity of transforming growth factor beta in a mouse model of breast cancer. Cancer Res 66: 6327–6335. 16778210 10.1158/0008-5472.CAN-06-0068 PMC1528715

[14] Nam JS, Terabe M, Mamura M, Kang MJ, Chae H, Stuelten C, et al (2008) An anti-transforming growth factor beta antibody suppresses metastasis via cooperative effects on multiple cell compartments. Cancer Res 68: 3835–3843. 10.1158/0008-5472.CAN-08-0215 18483268 PMC2587151

[15] Ho J, Kong JW, Choong LY, Loh MC, Toy W, Chong PK, et al (2009) Novel breast cancer metastasis-associated proteins. J Proteome Res 8: 583–594. 10.1021/pr8007368 19086899

[16] Xue T, Zhang Y, Zhang L, Yao L, Hu X, Xu LX (2013) Proteomic Analysis of Two Metabolic Proteins with Potential to Translocate to Plasma Membrane Associated with Tumor Metastasis Development and Drug Targets. J Proteome Res. 10.1021/pr301100r 23445495

[17] Ali NA, Molloy MP (2011) Quantitative phosphoproteomics of transforming growth factor-beta signaling in colon cancer cells. Proteomics 11: 3390–3401. 10.1002/pmic.201100036 21751366

[18] Souchelnytskyi S (2005) Proteomics of TGF-beta signaling and its impact on breast cancer. Expert Rev Proteomics 2: 925–935. 16307521 10.1586/14789450.2.6.925

[19] Aslakson CJ, Miller FR (1992) Selective events in the metastatic process defined by analysis of the sequential dissemination of subpopulations of a mouse mammary tumor. Cancer Res 52: 1399–1405. 1540948

[20] Halder SK, Beauchamp RD, Datta PK (2005) A specific inhibitor of TGF-beta receptor kinase, SB-431542, as a potent antitumor agent for human cancers. Neoplasia 7: 509–521. 15967103 10.1593/neo.04640 PMC1501161

[21] Laping NJ, Grygielko E, Mathur A, Butter S, Bomberger J, Tweed C, et al (2002) Inhibition of transforming growth factor (TGF)-beta1-induced extracellular matrix with a novel inhibitor of the TGF-beta type I receptor kinase activity: SB-431542. Mol Pharmacol 62: 58–64. 12065755 10.1124/mol.62.1.58

[22] Tanaka H, Shinto O, Yashiro M, Yamazoe S, Iwauchi T, Muguruma K, et al (2010) Transforming growth factor beta signaling inhibitor, SB-431542, induces maturation of dendritic cells and enhances anti-tumor activity. Oncol Rep 24: 1637–1643. 21042762 10.3892/or_00001028

[23] Masuda T, Tomita M, Ishihama Y (2008) Phase transfer surfactant-aided trypsin digestion for membrane proteome analysis. J Proteome Res 7: 731–740. 10.1021/pr700658q 18183947

[24] Rappsilber J, Mann M, Ishihama Y (2007) Protocol for micro-purification, enrichment, pre-fractionation and storage of peptides for proteomics using StageTips. Nat Protoc 2: 1896–1906. 17703201 10.1038/nprot.2007.261

[25] Boersema PJ, Raijmakers R, Lemeer S, Mohammed S, Heck AJ (2009) Multiplex peptide stable isotope dimethyl labeling for quantitative proteomics. Nat Protoc 4: 484–494. 10.1038/nprot.2009.21 19300442

[26] Muraoka S, Kume H, Watanabe S, Adachi J, Kuwano M, Sato M, et al (2012) Strategy for SRM-based verification of biomarker candidates discovered by iTRAQ method in limited breast cancer tissue samples. J Proteome Res 11: 4201–4210. 10.1021/pr300322q 22716024

[27] Narumi R, Murakami T, Kuga T, Adachi J, Shiromizu T, Muraoka S, et al (2012) A strategy for large-scale phosphoproteomics and SRM-based validation of human breast cancer tissue samples. J Proteome Res 11: 5311–5322. 10.1021/pr3005474 22985185

[28] Shiromizu T, Adachi J, Watanabe S, Murakami T, Kuga T, Muraoka S, et al (2013) Identification of missing proteins in the neXtProt database and unregistered phosphopeptides in the PhosphoSitePlus database as part of the Chromosome-centric Human Proteome Project. J Proteome Res 12: 2414–2421. 10.1021/pr300825v 23312004

[29] Matsubara T, Tanaka N, Krausz KW, Manna SK, Kang DW, Anderson ER, et al (2012) Metabolomics identifies an inflammatory cascade involved in dioxin- and diet-induced steatohepatitis. Cell Metab 16: 634–644. 10.1016/j.cmet.2012.10.006 23140643 PMC3496181

[30] Muraoka RS, Dumont N, Ritter CA, Dugger TC, Brantley DM, Chen J, et al (2002) Blockade of TGF-beta inhibits mammary tumor cell viability, migration, and metastases. J Clin Invest 109: 1551–1559. 12070302 10.1172/JCI15234 PMC151012

[31] Konicek BW, Dumstorf CA, Graff JR (2008) Targeting the eIF4F translation initiation complex for cancer therapy. Cell Cycle 7: 2466–2471. 18719377 10.4161/cc.7.16.6464

[32] Ramirez-Valle F, Braunstein S, Zavadil J, Formenti SC, Schneider RJ (2008) eIF4GI links nutrient sensing by mTOR to cell proliferation and inhibition of autophagy. J Cell Biol 181: 293–307. 10.1083/jcb.200710215 18426977 PMC2315676

[33] Yellen P, Chatterjee A, Preda A, Foster DA (2013) Inhibition of S6 kinase suppresses the apoptotic effect of eIF4E ablation by inducing TGF-beta-dependent G1 cell cycle arrest. Cancer Lett 333: 239–243. 10.1016/j.canlet.2013.01.041 23376634 PMC3640435

[34] Silvera D, Formenti SC, Schneider RJ (2010) Translational control in cancer. Nat Rev Cancer 10: 254–266. 10.1038/nrc2824 20332778

[35] Gilles C, Polette M, Zahm JM, Tournier JM, Volders L, Foidart JM, et al (1999) Vimentin contributes to human mammary epithelial cell migration. J Cell Sci 112 (Pt 24): 4615–4625. 10574710 10.1242/jcs.112.24.4615

[36] Nagaraja GM, Kaur P, Neumann W, Asea EE, Bausero MA, Multhoff G, et al (2012) Silencing Hsp25/Hsp27 gene expression augments proteasome activity and increases CD8+ T-cell-mediated tumor killing and memory responses. Cancer Prev Res (Phila) 5: 122–137. 10.1158/1940-6207.CAPR-11-0121 22185976 PMC3252476

[37] Jackson RJ, Hellen CU, Pestova TV (2010) The mechanism of eukaryotic translation initiation and principles of its regulation. Nat Rev Mol Cell Biol 11: 113–127. 10.1038/nrm2838 20094052 PMC4461372

[38] Nasr Z, Robert F, Porco JA Jr., Muller WJ, Pelletier J (2013) eIF4F suppression in breast cancer affects maintenance and progression. Oncogene 32: 861–871. 10.1038/onc.2012.105 22484424 PMC4863948

[39] De Benedetti A, Graff JR (2004) eIF-4E expression and its role in malignancies and metastases. Oncogene 23: 3189–3199. 15094768 10.1038/sj.onc.1207545

[40] de la Parra C, Otero-Franqui E, Martinez-Montemayor M, Dharmawardhane S (2012) The soy isoflavone equol may increase cancer malignancy via up-regulation of eukaryotic protein synthesis initiation factor eIF4G. J Biol Chem 287: 41640–41650. 10.1074/jbc.M112.393470 23095751 PMC3516715

[41] Silvera D, Arju R, Darvishian F, Levine PH, Zolfaghari L, Goldberg J, et al (2009) Essential role for eIF4GI overexpression in the pathogenesis of inflammatory breast cancer. Nat Cell Biol 11: 903–908. 10.1038/ncb1900 19525934

[42] Badura M, Braunstein S, Zavadil J, Schneider RJ (2012) DNA damage and eIF4G1 in breast cancer cells reprogram translation for survival and DNA repair mRNAs. Proc Natl Acad Sci U S A 109: 18767–18772. 10.1073/pnas.1203853109 23112151 PMC3503184

[43] Lou Y, Preobrazhenska O, auf dem Keller U, Sutcliffe M, Barclay L, McDonald PC, et al (2008) Epithelial-mesenchymal transition (EMT) is not sufficient for spontaneous murine breast cancer metastasis. Dev Dyn 237: 2755–2768. 10.1002/dvdy.21658 18773493

[44] Lamouille S, Derynck R (2007) Cell size and invasion in TGF-beta-induced epithelial to mesenchymal transition is regulated by activation of the mTOR pathway. J Cell Biol 178: 437–451. 17646396 10.1083/jcb.200611146 PMC2064840

[45] Das F, Ghosh-Choudhury N, Bera A, Kasinath BS, Choudhury GG (2013) TGFbeta-induced PI 3 kinase-dependent Mnk-1 activation is necessary for Ser-209 phosphorylation of eIF4E and mesangial cell hypertrophy. J Cell Physiol 228: 1617–1626. 10.1002/jcp.24327 23359369 PMC3855027

[46] Inman GJ, Nicolas FJ, Callahan JF, Harling JD, Gaster LM, Reith AD, et al (2002) SB-431542 is a potent and specific inhibitor of transforming growth factor-beta superfamily type I activin receptor-like kinase (ALK) recepotrs ALK4, ALK5, and ALK7. Mol Parmacol 62: 65–74. 10.1124/mol.62.1.65 12065756

[47] Hjelmeland MD, Hjelmeland AB, Sathornsumetee S, Reese ED, Herbstreith MH, Laping NJ, et al (2004) SB-431542, a small molecule transforming growth factor-beta-receptor antagonist, inhibits human glioma cell line proliferation and motility. Mol Cancer Ther 3: 737–745. 15210860

[48] Matsuyama S, Iwadate M, Kondo M, Saitoh M, Hanyu A, Shimizu K, et al (2003) SB-431542 and Gleevec inhibit transforming growth factor-beta-induced proliferation of human osteosarcoma cells. Cancer Res 63: 7791–7798. 14633705

