# Supplementary material for: Differential Proteome Analysis Identifies TGF-β-Related Pro-Metastatic Proteins in a 4T1 Murine Breast Cancer Model
Source: PLoS One. 2015 May 18;10(5):e0126483. doi: 10.1371/journal.pone.0126483 (PMC4436378; doi:10.1371/journal.pone.0126483)
Supplement: S1 Table — Lung metastases samples from untreated and treated with SB-431542, were subjected to iTRAQ labeling and LC-MS/MS analysis to profile their expression levels. The list of protein ranking, based on the OPLS-DA (Fig 4B), indicates the highest confidence and greatest contribution separation between the untreated and SB-431542-treated mice, p[1]>0.02. Their expression ratios, control/ SB-431542, represent a significant downward trend in mean value ((p(corr)> 0.80, n = 4 per group). (DOCX) [file pone.0126483.s005.docx]

**S1 Table.**
