# Supplementary material for: Differential Proteome Analysis Identifies TGF-β-Related Pro-Metastatic Proteins in a 4T1 Murine Breast Cancer Model
Source: PLoS One. 2015 May 18;10(5):e0126483. doi: 10.1371/journal.pone.0126483 (PMC4436378; doi:10.1371/journal.pone.0126483)
Supplement: S2 Table — (DOCX) [file pone.0126483.s006.docx]

**S2 Table.**

**PCR primer sequences.** The primer set used in this study:

| **Gene name** | **Primer sequences** |
| --- | --- |
| 18S_forward | ATT GGA GCT GGA ATT ACC GC |
| 18S_reverse | CGG CTA CCA CAT CCA AGG AA |
| Serpine1_forward | GCC AGG GTT GCA CTA AAC AT |
| Serpine1_reverse | GCC TCC TCA TCC TGC CTA A |
| Vimentin_forward | TCC ACT TTC CGT TCA AGG TC |
| Vimentin_reverse | AGA GAG AGG AAG CCG AAA GC |
| eEF2_forward | CGG ATG TTG GCT TTC TTG TC |
| eEF2_reverse | GCT TCC CTG TTC ACC TCT GA |
| eIF4G1_forward | TCTTCCTTCACCACGTCCTC |
| eIF4G1_reverse | CTCCAGGCCCTTGTAGTGAC |
| eIF4E_forward | TCTGGGTTAGCAACCTCTTGA |
| eIF4E_reverse | TCTAAGATGGCGACTGTGGA |
| eEF1A1_forward | CCTTTCCCATTTTTGCTTTG |
| eEF1A1_reverse | AGTCGCCTTGGACGTTCTTT |
| eIF2S3_forward | TTGGCTTAATGGTCACTCCTC |
| eIF2S3_reverse | CCGTGTGCACAGAAGTAGGA |
| eIF4A1_forward | GCTATCCACAATCTCGTTCCA |
| eIF4A1_reverse | TCATGTCTGCGAGTCAGGAT |
